# Urethral duplication type influences on the complications rate and number of surgical procedures

**DOI:** 10.1590/S1677-5538.IBJU.2016.0269

**Published:** 2017

**Authors:** Roberto Iglesias Lopes, Amilcar Martins Giron, Marcos Figueiredo Mello, Cristovao Machado Barbosa, Joana dos Santos, Paulo Renato Marcelo Moscardi, Victor Srougi, Francisco Tibor Denes, Miguel Srougi

**Affiliations:** 1 Unidade de Urologia Pediátrica, Divisao de Urologia, Hospital das Clinicas, Faculdade de Medicina da Universidade de São Paulo, Brasil; 2 Division of Urology, The Hospital for Sick Children, University of Toronto, Canada

**Keywords:** Surgical Procedures, Operative, Urethra, complications [Subheading]

## Abstract

**Introduction:**

Urethral duplication is rare. Characterized by the presence of two urethral channels. This anomaly presents a great variety of clinical findings that depend on the type of duplication that often is associated with other anomalies.

**Material and Methods:**

We report thirteen boys with urethral duplication managed in our institution between 1988-2015. Clinical findings, associated anomalies, treatment of urethral duplication and our results are described. Patients were classified according to Effmann classification.

**Results:**

Mean patient's age was 38.3±34.7 months (3-136 months). Mean follow-up was 7.7±3.4 years (3y8m-14y2m). Type II A2 was the most common pattern (8/13 patients, 61.5%), followed by type IA (3/13 patients, 23%) and IIA1 (2/13 patients, 15.3%). The most frequent clinical manifestations were urinary tract infections (UTI) observed in 11/13 patients (84.6%) and anal urinary leakage, found in 7/13 patients (53.8%). Associated anomalies were found in 9/13 patients (69.2%).

Required surgeries were 3.53±2.84 procedures per patient. Considering groups: Type IIA2 4.25±3.28, type IIA1 4±1.41 and type IA 1.33±0.57 needed procedures per patient. Complications rate were 0% for type IA, 50% for type IIA1 and 75% for type IIA2.

**Conclusions:**

Patients with incomplete duplication (type I A or I B) can totally be asymptomatic, with no need of surgical correction. Type IIA2 is the most complex form of duplication to correct and multiple procedures might be required because of the very hypoplastic orthotopic dorsal urethral tissue. Surgical treatment should be individualized and parents should be advised on complications and need of multiple surgeries according to urethral duplication type.

## INTRODUCTION

Urethral duplication is a rare congenital anomaly characterized by two urethral channels, whose location and extension present variations. It is more common in males, occurring usually in the sagittal plane. In females, this anomaly is rare and most often associated with bladder duplication (1-4).

Because of the diversity of clinical manifestations, diagnosis is difficult, as well as its classification. Patients can be either asymptomatic or symptomatic, most common clinical findings being incontinence, obstruction, recurrent urinary infection, and occasionally double urinary stream (1-4).

The objective of this study was to review our experience in the management of urethral duplication anomalies and to determine whether the type of urethral duplication influences on the number of surgical procedures needed for repair and complication rates.

## MATERIALS AND METHODS

Medical records of patients with urethral duplication anomalies were analyzed retrospectively. We searched in our hospital database for urethral duplication cases submitted to surgical treatment. Urethral duplication cases without surgical management were not included in this study. Clinical characteristics such as age at presentation, type of urethral duplication, clinical findings, associated anomalies, surgical treatment, complications and results were reviewed. Patients were classified according to Effmann et al., as shown in Table-1 (5).

**Table 1:** Classification of urethral duplication (Effmann et al., 1976).

Type I		Incomplete urethral duplication (accessory urethra)
	A - Distal:	The meatus is on the dorsal or ventral surface, but it does not have communication with the urethra or the bladder.
	B - Proximal:	The accessory urethra originates from the normal urethra and ends blindly.
Type II		Complete Duplication
	A - Two meatus:	1 - two non-communicating urethras originating independently from the bladder
		2 - the second urethra originates from the first, with an independent channel until the second meatus
	B - One meatus:	1 - two urethras originate from the bladder or of the subsequent urethra joining later in a single channel
Type III		Urethral duplication, part of a complete or incomplete caudal duplication

Thirteen male patients with urethral duplication were managed surgically at our institution between 1988 and 2016. All patients were carefully assessed preoperatively by clinical history, physical examination, kidney and bladder ultrasonography and voiding cystourethrography (VCUG). For surgical planning, an urethrocystoscopy was performed at the beginning of the operation to aid the surgical decision.

Uroflowmetry and urodynamics studies were only performed in cases of associated dysfunctional voiding (irritative or obstructive lower urinary tract symptoms). Follow-up was done by regular clinic visits (every 6 months to 1 year) that included physical examination and ultrasonography of urinary system. VCUG was carried out only if there was recurrent urinary tract infection or suspicion of urethral obstruction.

After collection of analytical data, urethral duplication type was correlated to the number of surgical procedures needed for repair and complications rates.

### Statistical analysis

All values were presented as mean ± standard deviation with the ranges. Statistical analysis was done using ANOVA (Bonferroni) for categorical comparisons. Results were considered significant when p value was equal or less than 0.05.

## RESULTS

Mean age at surgical intervention was 38.3±34.7 months (range: 3 to 136 months). Mean age±standard deviation (range) was 72.3±55.1 (38 to 136 months) for group IA, 43±26.8 (24 to 62 months) for group IIA1 and 24.3±19.3 (3 to 61 months) for group IIA2. Mean follow-up was 7.7±3.4 years (3y8m-14y2m).

Type IIA2 was the most common pattern, found in 8/13 patients (61.5%), followed by type IA (3/13 patients, 23%) and IIA1 (2/13 patients, 15.3%). The most frequent clinical manifestations were urinary tract infections (UTI) observed in 11/13 patients (84.6%) and anal urinary leakage, found in 7/13 patients (53.8%). Associated anomalies were found in 9/13 patients (69.2%). The most common associated anomalies were vesicoureteral reflux (6/13 patients, 46%) and renal abnormalities (4/13 patients, 30.7%), as demonstrated in Table-2.

**Table 2:** Clinical features of our cohort.

Pt	Age	Effmann	Clinical finding	Associated anomalies	Treatment # 1	Treatment # 2	Treatment # 3	Treatment # 4	Treatment #5	Complications	Followup
1	3y2m	IA	Hypospadic subcoronal urethral meatus	No abnormalities	Ventral incision in the accessory urethra	Denis Browne technique (hypospadia repair)				No	6y6m
2	11y4m	IA	UTI +purulent discharge from epispadic meatus	Left VUR grade III	Accessory urethral resection					No	3y8m
3	3y7m	IA	UTI + purulent discharge from epispadic meatus	No abnormalities	Accesory urethral resection					No	5y1m
4	2y	IIA1	Anal urinary leakage + UTI	No abnormalities	Vesicostomy	Perineal urethrostomy	Second perineal urethrostomy + vesicostomy closure	Preputial island flap neourethroplasty	2 nd stage urethroplasty	Urethral stenosis (treated successfully)	7y11m
5	5y2m	IIA1	Anal urinary leakage + UTI	Tetralogy of Fallot/ Left solitary kidney / Absence of the second right costal arch / Hemivertebra between the fourth and fifth lumbar vertebrae	Colostomy performed in another institution	Perineal urethrostomy + colostomy closure	Preputial island neourethroplasty			No	12y
6	1y6m	IIA2	Anal urinary leakage + UTI	Posterior urethral valves / Right VUR grade III / Right solitary kidney	Endoscopic transurethral valve resection	Resection of the accessory urethra + urethral stenosis dorsal urethra	T-T anastomosis dorsal urethra			No	6y3m
7	5m	IIA2	Anal urinary leakage + UTI	Left kidney exclusion / Right vesicoureteral reflux grade IV	Vesicostomy	Resection of the accessory urethra + urethral stenosis dorsal urethra	Urethrotomy + Neourethroplasty (distal correction + meatoplasty)			Urethral stenosis (treated successfully)	14y2m
8	5y1m	IIA2	UTI	Anal imperfuration / Left vesicoureteral reflux grade V / Spina Bifida	Vesicostomy	Left nephrectomy + cystostomy	Bladder augmentation + Monti			Urethral stenosis + low capacity bladder (treatment: augmentation and Monti)	4y9m
9	2y4m	IIA2	Anal urinary leakage + UTI	Right dysplastic kidney / Left vesicoureteral reflux grade III/ Congenital megacolon	Vesicostomy + sigmoidostomy	Perineal urethrostomy	Preputial island neourethroplasty	Urethrotomy	Preputial neourethroplasty	Urethral stenosis (treated successfully)	10y3m
10	3y3m	IIA2	Anal urinary leakage + UTI	Right ectopic crossed fused kidney	8 surgeries to correct the urethra/bladder in another institution	Vesicostomy	Perineal urethrostomy	Prepuce urethroplasty + vesicostomy closure + cystostomy	Urethral transplantation of acellular matrix	Urethral stenosis (treated successfully)	8y5m
11	2y5m	IIA2	Anal urinary leakage	No abnormalities	Perineal urethrostomy	Preputial island neourethroplasty				No	5y4m
12	3m	IIA2	UTI (sepsis)	Bilateral ureteral duplication	Cystostomy	Vesicostomy	Vesicostomy closure (obstruction)	Mitrofanoff		Urethral stenosis (ended up with Mitrofanoff)	12y5m
13	1y	IIA2	Multiple UTI	Anal imperforation / Left vesicoureteral reflux grade IV	Vesicostomy	Bladder augmentation + Mitrofanoff				Urethral stenosis + low capacity bladder (treatment: augmentation and Mitrofanoff)	11y7m

Required surgeries were 3.53±2.84 procedures per patient (1 to 12 procedures). Considering groups: type IA needed 1.33±0.57 procedures per patient (1 to 2 surgeries), type IIA1 needed 4±1.41 procedures per patient (3 to 5 surgeries) and type IIA2 need 4.25±3.28 procedures per patient (2 to 12 surgeries). Children with types IIA1 and IIA2 of urethral duplication underwent significantly more surgical procedures than type IA (p values 0.05 and 0.05, respectively). Complications rate were 0% for type IA, 50% for type IIA1 (1/2 patients had urethral stenosis) and 75% for type IIA2 (6/8 patients, with 6/8 developing urethral stenosis and 2/8 with a defunctionalized bladder that required augmentation). However, no statistically significant difference was found when number of surgeries for types IIA1 and IIA2 were compared (p=1.0)

## DISCUSSION

Urethral duplication is a rare anomaly, with great diversity of clinical presentations. Some explanations of its morphogenesis have been proposed and as a consequence, several hypotheses were formulated aiming to explain failures in its embryogenesis.

The occurrence of urethral duplication with the accessory urethra epispadic, as in cases 2 and 3 can be associated with the same embryology theory of the exstrophy-epispadias complex, in which it might occur failure of the fusion of lateral mesoderm in the midline, between the ectoderm and the endoderm of the cloacal membrane (5).

In cases of duplication in which one of the urethral meatus is located in the anal or perineal region (cases 4 to 13), these are probably secondary to failure of the urorectal septum normal development (6).

In the type III urethral duplication, associated with the syndrome of caudal duplication, that can present duplications of the uterus, vagina, rectum, colon, among others, the morphogenetic defect occurs due to the division of the notochord in earlier phase of the embryonic development (5). In cases of collateral urethral duplication, that is, when both urethras are side by side, the urethral groove could be divided before forming the urethra in the medium line, originating two urethras (7).

In this study, Effmann et al. (5) classification was used because it is considered the most complete, as described in Table-1.

Patients’ clinical findings with urethral duplication are variable, depending on the duplication type. According to Bogaert, (8) an accessory urethra ends blindly (type I A) and can cause few symptoms as elimination of purulent secretion or be asymptomatic. Type IB can also be asymptomatic, many times difficult of being differentiated of urethral diverticulum. In cases of duplication with epispadic accessory urethra, a dorsal curvature of the penis can occur; in the cases of hypospadic accessory urethra, ventral curvature can occur.

Types I B and IIB1 can be asymptomatic (8). Probably this is the reason why these are considered rare (difficult diagnosis due to the lack of clinical manifestations).

In cases of complete duplication, types IIA1 and IIA2, the most common clinical manifestations include recurrent urinary tract infections, double urinary stream and urinary obstruction. Type IIA2, with a perineal urethra or in the anal channel, also called duplication in Y or H, usually presents a more functional ventral urethra. In those children, clinical findings may include urine elimination along with stool. However, in this presentation, some patients can present normal dorsal orthotopic urethra also called congenital urethroperineal fistula. It is considered as a separated entity of the urethral duplications by some authors.

Some patients with complete duplication can present urinary incontinence. Gross and Moore reported this manifestation in seven of 19 examined patients. In our cases, no patient presented urinary incontinence. In most of the cases, the ventral urethra crosses the sphincter, also containing the accessory glands and the verumontanum (3). These patients can also present stress urinary incontinence. This clinical manifestation is due to the accessory urethra, which is not usually surrounded by the urinary sphincter (9).

In cases of collateral duplication, the double flow is the most common clinical manifestation. These patients usually present several other associated congenital anomalies, such as colon and bladder duplications, hemivertebrae, dysplasia and renal agenesis, among others (9).

No study about fertility and ejaculation in patients with urethral duplication was reported in the literature. The association between urethral duplication and posterior urethral valves was described by Lorenzo et al., (10) Fernbach et al. (11) and Ramanujam et al. (12). This association presents more difficulty to provide an accurate diagnosis of urethral duplications, as verified in case 6.

Other anomalies associated with urethral duplication include bifid scrotum, cryptorchidism, hypospadias, megalourethra, micropenis, vesicoureteral reflux, agenesis and renal ectopy, dysplastic-multicystic kidney, vertebral anomalies (sacral agenesis, thoracic hemivertebra), anorectal anomalies, trachea-esophagic fistula and penile, vagina, uterus, bladder and colon duplications (1, 2, 6, 7, 13).

The diagnosis of urethral duplication is performed by clinical history, physical exam and imaging methods, especially voiding cystourethrography. Kidney and bladder ultrasonography is recommended to investigate associated anomalies. Urethrocystoscopy is important for surgical planning. Magnetic resonance urography or excretory urography might be useful to further depict upper tract abnormalities.

Treatment of this anomaly depends on the duplication type and its clinical manifestations. Before any surgical decision, it must be identified which urethra is more functional. Patients with incomplete duplication (type I A or I B) can totally be asymptomatic, with no need of surgical correction. If those patients present purulent secretions or local infection, the accessory urethra should be resected (8). Other option is the opening of the ventral wall of the accessory hypospadic urethra and posterior neourethroplasty, as described by Podesta et al. (14) to treat patients with hypospadias and incomplete urethral duplication. In these cases, it was observed fewer surgeries to proper surgical repair and no complications in our cohort.

**Figure 1:**
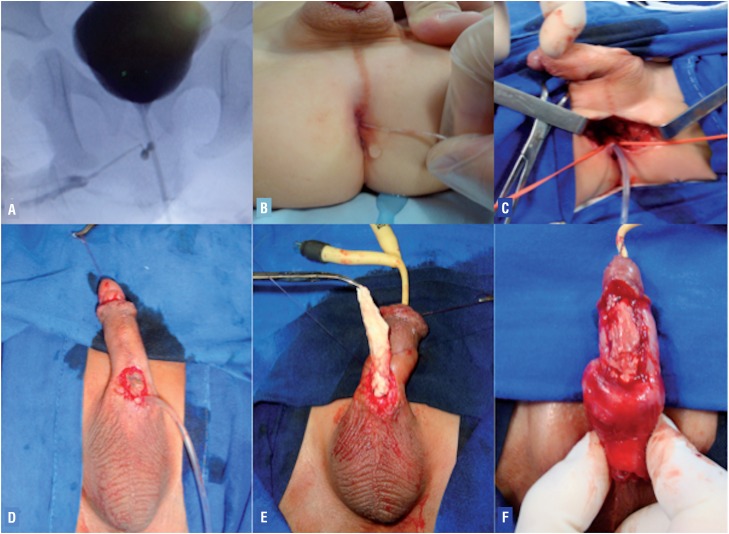
A) Voiding cystourethrogram revealed urethral duplication with a narrow cal-iber dorsal penile orthotopic urethra and the ventral urethra meatus opening in the rec-tum (type IIA urethral duplication); B) catheterization of the ventral urethra by its opening in the rectum (type IIA urethral duplication); c) Intraoperative picture showing dissection of the ventral urethra; D) Intra-operative view depicting catheterization of the accessory urethral meatus located at penoscrotal junction; E) Onlay acellular matrix urethral transplantation; F) final aspect of onlay acellular matrix urethral transplantation (patient 10 of Table-2).

In cases where the patient presents normal dorsal urethra and ventral urethra interfering in the anal canal (anorectal junction) or in the rectum (urethroperineal fistula) - type IIA2, our surgical approach aimed the initial urethrostomy of the ventral urethra and preservation of the dorsal orthotopic urethra. After that, a neourethroplasty using flaps or grafts was usually performed. In difficult redo cases, even an acellular matrix transplantation was performed for one of our patients (case 10) as shown in Figure 1.

These patients with urethral duplication of hypospadic type, in which the ventral urethra is more functional and located in the perineal or anal area (type IIA2), are challenging to surgical correction and more commonly they present complications such as neourethral dehiscence and stenosis, which are common in these neourethroplasty types. In our study, a significant rate of surgical procedures per patient and complications were observed for type IIA duplications. Type IIA1 needed 4±1.41 procedures per patient (3 to 5 surgeries) and type IIA2 need 4.25±3.28 procedures per patient (2 to 12 surgeries) and rate of complications were 50% for type IIA1 and 75% for type IIA2 which should be informed for patients and families preoperatively. Type IIA2 is the most complex form of duplication to correct, and in such cases the orthotopic urethra usually has an extensive hypoplastic segment. Hence, it is recommended to mobilize extensively the ventral functional urethra to the perineal-scrotal junction to prevent complications and anticipate that multiple procedures might be required because of the very hypoplastic orthotopic dorsal urethral tissue.

## CONCLUSIONS

Urethral duplication is a rare anomaly, with several forms of clinical presentation, often accompanied by other anomalies, and sometimes with difficult diagnosis. The treatment of urethral duplication should be individualized, according to its type. Significantly higher rates of surgical procedures per patient and, possibly complication rates were observed for type IIA duplications, which should be informed for patients and families preoperatively.

## References

[1] Prasad N, Vivekanandhan KG, Ilangovan G, Prabakaran S (1999). Duplication of the urethra. Pediatr Surg Int.

[2] Salle JL, Sibai H, Rosenstein D, Brzezinski AE, Corcos J (2000). Urethral duplication in the male: review of 16 cases. J Urol.

[3] Onofre LS, Gomes AL, Leão JQ, Leão FG, Cruz TM, Carnevale J (2013). Urethral duplication-a wide spectrum of anomalies. J Pediatr Urol.

[4] Coleman RA, Winkle DC, Borzi PA (2010). Urethral duplication: cases of ventral and dorsal complete duplication and review of the literature. J Pediatr Urol.

[5] Effmann EL, Lebowitz RL, Colodny AH (1976). Duplication of the urethra. Radiology.

[6] deVries PA, Friedland GW (1974). Congenital “H-type” ano-urethral fistula. Radiology.

[7] Kennedy HA, Steidle CP, Mitchell ME, Rink RC (1988). Collateral urethral duplication in the frontal plane: a spectrum of cases. J Urol.

[8] Bogaert GA, Gearhart JP, Rink RC, Mouriquand PDE (2001). Urethral duplication and other urethral anomalies. Pediatric urology.

[9] Atala A, Marshall FF (1996). Congenital urethral duplication. Textbook of operative urology.

[10] Lorenzo RL, Turner WR, Bradford BF, Upshur J, Sexton FM (1981). Duplication of the male urethra with posterior urethral valves. Pediatr Radiol.

[11] Fernbach SK, Maizels M (1984). Posterior urethral valves causing urinary retention in an infant with duplication of the urethra. J Urol.

[12] Ramanujam TM, Sergius A, Usha V, Ramanathan S (1998). Incomplete hypospadiac urethral duplication with posterior urethral valves. Pediatr Surg Int.

[13] Savanelli A, Schiano A, Esposito C, Russo S, Dolezalova H (1998). Congenital megalourethra associated with urethral duplication and imperforate anus. Pediatr Surg Int.

[14] Podesta ML, Medel R, Castera R, Ruarte AC (1998). Urethral duplication in children: surgical treatment and results. J Urol.

